# Osmotically assisted reverse osmosis, simulated to achieve high solute concentrations, at low energy consumption

**DOI:** 10.1038/s41598-022-16974-x

**Published:** 2022-08-12

**Authors:** Behzad H. M. Beigi, Siddharth Gadkari, Jhuma Sadhukhan

**Affiliations:** Department of Chemical and Process Engineering, Centre for Environment and Sustainability, University of Surrey, Guildford, GU2 7XH UK

**Keywords:** Chemical engineering, Pollution remediation

## Abstract

Microbial electrosynthesis (MES), is an emerging technology, for sustainable wastewater treatment. The dilute acetate solution, produced via MES, must be recovered, as dilute solutions can be expensive to store and transport. The acetate is expensive and environmentally damaging to recover by heat-intensive evaporative methods, such as distillation. In pursuit of a better energy economy, a membrane separation system is simulated to raise the concentration from 1 to 30 wt%, at a hydraulic pressure of approximately 50 bar. The concentrate is then simulated to be heat dried. Reverse osmosis (RO) could rase the acetate concentration to 8 wt%. A novel adaptation of osmotically assisted reverse osmosis (OARO) is then simulated to increase the concentration from 8 to 30 wt%. The inclusion of OARO, rather than a standalone RO unit, reduces the total heat and electric power requirement by a factor of 4.3. It adds to the membrane area requirement by a factor of 6. The OARO simulations are conducted by the internal concentration polarisation (ICP) model. Before the model is used, it is fitted to OARO experimental data, obtained from the literature. Membrane structure number of 701 µm and permeability coefficient of 2.51 L/m^2^/h/bar are ascertained from this model fitting exercise.

## Introduction

Microbial electrosynthesis (MES), is an emerging wastewater treatment technology, were acetic acid (AA) is the most widely studied by-product^2,19,36^. Gadkari et al.^20^ investigated MES, for renewable production of acetate, by consuming waste carbon dioxide, produced from other processes. The greatest obstacle against this adaptation of the MES process was found to be the low product concentrations, especially under continuous operation mode^10,48^. The acetate recovery under such low concentrations was found to be unviable. Furthermore, the produced AA is under investigation as a substrate for MES processes, to produce fuel and other expensive products^21^. The unused substrate must be removed, to meet environmental consents.

The separation of AA from water is complicated, expensive and environmentally burdensome^42,62,64^. If a mole of sodium hydroxide is added for every mole of AA in the solution, sodium acetate salt is formed, which is far less permeable than AA. Therefore, it can be separated via reverse osmosis (RO), much more efficiently. The produced salt is marketable, it is more expensive than acetic acid, and it can be converted back into its corresponding volatile fatty acid and alcohol. 1 wt% sodium acetate solution is assumed as an optimistic, yet realistic concentration for the feed to the separation system, investigated here.

Distillation and heat drying are among widely used separation techniques, in process industries. Such heat-intensive separation systems have been deemed viable, partially due to the availability of low-cost, non-renewable heat. More energy-efficient separation technologies, that can be powered renewably, are preferred, on both economic and environmental grounds. For concentrating aqueous solutions, an example of a suitable technique is a renewably powered membrane separation system. Such systems are often many times more energy efficient than evaporative alternatives, due to water’s unusually high latent heat of evaporation.

When a semi-permeable membrane is placed between two solutions of different molar concentrations, water permeates from the lower concentration side of the membrane to the higher concentration side. This phenomenon is referred to as forward osmosis (FO). The flux of water across the membrane can be obstructed by applying adequate hydraulic pressure against the osmotic flux of water. The hydraulic pressure difference across the membrane resulting in zero water flux is osmotic pressure difference between the two solutions. If the hydraulic pressure, against the natural flux, exceeds the osmotic pressure difference, water permeates from the higher to the lower concentration side of the membrane. This phenomenon, known as reverse osmosis (RO), is widely used to recover water from aqueous solutions and concentrate the solute.

Potential advantages of RO over thermal evaporation include: (1) significantly lower power consumption, (2) avoiding air contamination and (3) avoiding thermally induced chemical reactions^23,29,59^. The liquid at the low concentration side of the RO membrane is referred to as the “Permeate”, and the concentrated solution, is referred to as “Retentate”.

The goal in this paper is to achieve high concentrations, osmotically. Achieving, for example, 30 wt% sodium acetate, via RO would require at least 182 bar pressure (see “Water flux model for RO” section). This would breach the design pressure of RO membranes, many times over. However, the hydraulic pressure requirement can be reduced by assisting the RO, with osmotic pressure, via dosing a solute into the permeate side of the RO membrane. This phenomenon is referred to as osmotically assisted reverse osmosis (OARO). The driving force for conventional RO is hydraulic pressure, whilst the driving force for OARO is a combination of hydraulic pressure and osmotic pressure. Table 1, summarises these membrane systems and highlights their distinctions.

**Table 1:** Membrane system classification, based on the driving force.

Membrane technology	Abbreviation	Driving force for water flux	The membrane system’s work power (net)
Reverse osmosis	RO	Hydraulic pressure	Work is consumed by the membrane system
Pressure retarded osmosis	PRO	Osmotic pressure	Work is produced by the membrane system
Forward osmosis	FO	Osmotic pressure	Zero work due to zero hydraulic pressure difference
Osmotically assisted reverse osmosis	OARO	Osmotic and hydraulic pressures	Work is consumed by the membrane system

OARO is a recent field of research, which has gained substantial traction in the past two years. There is ample opportunity to research toward novel applications, designs and models, on this topic. However, there has been a number of highly innovative publications on this topic, already. Peters and Hankins^47^ proposed osmotic assist by dissolving carbon dioxide and ammonia gasses into the permeate water. These gases are recovered and reused via a boiler and condenser system.

Others investigated OARO for clean water recovery^4,6,7,46^. Baena-Moreno et al.^5^ developed an energy efficient integration of FO with OARO, for recovering minerals from acid mine drainage fluid. They achieved high water purity, at reduced energy consumption.

Togo et al.^57^ and Nakagawa et al.^40^ investigated co-current flow in the permeate and retentate side of the membrane. The counter-current flow provides a more efficient separation. Therefore, Chen and Yip^16^ innovated a counter current flow OARO system, termed cascading osmotically mediated reverse osmosis (COMRO). Here, the osmotic assist is provided by the feed rather than the product, which limits the concentration that could be attained.

Bouma and Lienhard^12^ and Mo et al.^38^ investigated split-feed counterflow OARO, in which some of the feed is fed to the retentate side and the rest is fed to the permeate side, to provide osmotic assist. The provision of osmotic assist via the split-feed stream, rather than the split-retentate, limits the achievable product concentration. Bouma and Lienhard^12^ recommended repeating the above process to further concentrate the solution.

Blohm et al.^11^ patented a different adaptation of OARO, where osmotic assist is provided by splitting the retentate, instead of the feed. Under this split-retentate mode, the osmotic assist can be raised by adding to the OARO membrane area. In contrast, under split-feed mode, the osmotic assist is fixed, as dictated by the feed concentration.

In this paper a novel adaptation of OARO is simulated, which combines the qualities of the two designs adopted by Bouma and Lienhard^12^ and Blohm et al.^11^. Here, high concentration is achieved by the split-retentate counter-current system adopted by Blohm et al.^11^. However, osmotic potential waste is avoided, by merging the recycled draw solution with a stream of matching concentration, as achieved by Bouma and Lienhard^12^.

In “Methodology” section, mathematical models for RO and OARO simulation are described. In “Novel RO separation scheme” section, the novel design is described, in detail. In “Results and discussions” section, the models are fitted to experimental data, and used to simulate the novel design.

## Methodology

Here, a model is laid out, to describe the flux of water through the membrane, at any point within the membrane module. The flux model can then be used, to predict a flux profile throughout the flow path of a module, from which the performance of a module is simulated.

### Water flux model for RO

The osmotic pressure at each side of the membrane, $\pi$ is estimated from the van’t Hoff equation^52^, using $R$ as the ideal gas constant, $T$ as the absolute temperature, $C$ as the molar concentration, and $i$ as the number of ions associated with every mole of dissolved solid equivalent, as shown in Eq. (1). For example, $i$ is equal to one for glucose, two for sodium acetate and three for sodium sulphate.

1 $$\pi =iRTN/V=iRTC$$

The osmotic pressure difference, across a membrane skin, $\Delta \pi$ can then be estimated, as shown in Eq. (2). Here, the subscript $m$ represents the membrane skin surfaces, on both sides, and the subscripts $h$ and $l$ represent the higher concentration and lower concentration sides of the membrane.

2 $$\Delta \pi =iRT\left({C}_{m,h}-{C}_{m,l}\right)$$

The water flux, $J_{w}$ can be estimated, as shown in Eq. (3), using $\Delta P$ as the hydraulic pressure difference, across the membrane, and $A_{M}$ as the water permeability coefficient of the membrane^15,39,60^.

3 $$J_{w} = A_{M} \left( {\Delta P - \Delta \pi } \right)$$

The diffusive flux of the solute, away from the membrane, at the retentate side ($J_{h}$) is described by a liquid film mass transfer model, as shown in Eq. (4), where $C$ is molar concentration in mol/m^3^, and $k$ is the mass transfer coefficient in m/s^26^. Also, the subscripts $m$, $b$ and $h$ refer to the membrane interface, the liquid bulk and the retentate side of the membrane, respectively.

4 $$J_{h} = k_{h} \left( {C_{m,h} - C_{b,h} } \right)$$

$J_{w}$ is the volumetric flux of water in units of m/s, forced from the retentate side to the permeate side of the membrane, whilst $J_{h}$ is the molar diffusive flux of the solute, in units of mol/m^2^/s, in the opposite direction to $J_{w}$.

Under steady-state regime, there is no accumulation in the liquid film. At the retentate side, the rate at which ions are prevented to pass is equal to the rate at which they diffuse out of the liquid film, as shown in Eq. (5)^35^.

5 $$J_{h} = J_{w} C_{b,h}$$

Equations 2, 3, 4 and 5 can be used to derive Eq. (6), to predict the water flux in reverse osmosis. This equation excludes $\Delta \pi$,$C_{m,h}$ and $J_{h}$. Instead, it is a function of only two variables $C_{b,h}$ and $\Delta P$, which are tangible and easy to measure.

6 $$J_{w} = A_{M} k_{h} \left( {\frac{{\Delta P - iRTC_{b,h} }}{{k_{h} + A_{M} iRTC_{b,h} }}} \right)$$

Here, flux is approximated to occur in a single length dimension, perpendicular to the membrane surface. This is a widely adopted assumption, for modelling mass transfer through thin layers, and it is referred to as film theory, in chemical engineering literature^9^.

### Water flux model for OARO

**Figure 1:**
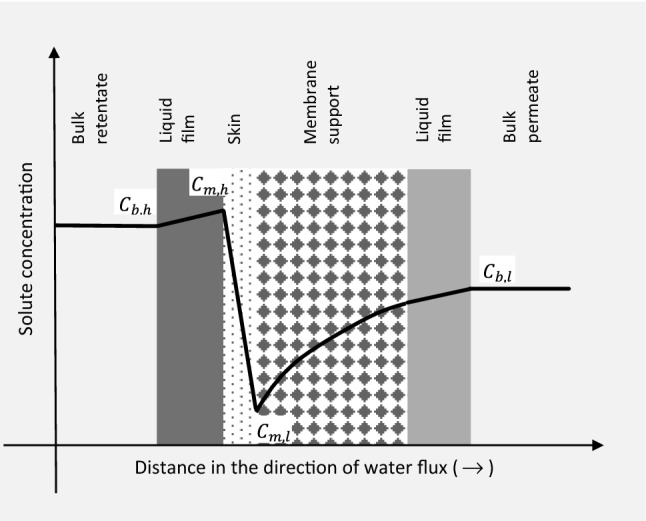
Schematic concentration profile depicted for OARO membranes^44^.

The solute concentration profile, from the retentate side to the permeate side, is depicted in Fig. 1, for osmotically assisted reverse osmosis (OARO). Here, unlike RO, there is a substantial concentration gradient at the support medium.

Since this concentration gradient cannot be accurately accounted for, by Eq. (4), Park et al.^44^ recommended the internal concentration polarization (ICP) model, as shown in Eq. (7). Here, $C_{b,l}$ is the bulk permeate concentration and $B$ is the salt permeability. $K$ is a constant described by Eq. (8), where $\delta_{s}$ is the thickness, $\tau$ is the tortuosity, and $\upvarepsilon$ is the porosity of the porous support layer, and $D$ is the solute diffusion coefficient, in water. The rest of the parameters, in Eq. (7) are as defined, previously.

7 $$\frac{{\Delta P - J_{w} /A_{M} }}{iRT} = \frac{{C_{b,h} exp^{{\left( {J_{w} /k_{h} } \right)}} - C_{b,l} exp^{{\left( { - J_{w} K} \right)}} }}{{1 + B\left( {exp^{{\left( { - J_{w} K} \right)}} - 1} \right)/J_{w} }}$$

8 $$K = \frac{{\tau \delta_{s} }}{{D\upvarepsilon }}$$

### Empirical mass transfer coefficient calculations

The simulation of the water flux in both the RO and the OARO cases, requires mass transfer coefficient at the retentate side, $k_{h}$. This constant can be determined, by model fitting of Eq. (6) to a range of measured flux values and their corresponding hydraulic pressure and bulk solution concentrations, in an RO unit.

$k_{h}$ can also be calculated using the empirical film-model correlation, demonstrated by Strathmann^55^, as described next. First, Reynolds number $N_{Re}$, is calculated for the liquid flow in the membrane channels, according to Eq. (9), using $\rho$ as the liquid density, $\mu$ and the liquid dynamic viscosity, $v$ as the superficial velocity and $d_{H}$ as the size of the flow channels.

9 $$N_{Re} = \frac{{\rho d_{H} v}}{\mu }$$

The Schmidt number, $N_{Sc}$ is described in Eq. (10), using $D$ as the diffusion coefficient of the aqueous ion. The diffusion coefficient of acetate (1.089 × 10^–9^ m^2^/s) is used in all calculations Buffle et al.^14^. It is slightly less than that of the sodium cations, making it the rate-limiting diffusion coefficient.

10 $$N_{Sc} = \frac{\mu }{\rho D}$$

Upon calculation of the Reynolds number and the Schmidt number, the Sherwood number can be calculated for all Reynolds numbers smaller than 2100, according to Eq. (11), using $L$ as the length of the flow channel, which is the length of the module, in this case.

11 $$N_{Sh} = 1.62N_{Re}^{0.33} N_{Sc}^{0.33} \left( {d_{H} /L} \right)^{0.33}$$

The liquid film mass transfer coefficient can be calculated from Eq. (12).

12 $$k = DN_{Sh} /d_{H}$$

At the retentate side, the resistance to mass transfer is attributed to liquid film, entirely ($k_{h} = k$).

### Axial profiles for flux, concentrations and flow rates

The concentration changes along the length of the membrane module, due to the water flux in or out of the flow channels. The flux changes due to the changes in concentration. Also, there is a small pressure drop through the flow path. Park et al.^44^ listed the following equations to provide profiles along the flow paths, for concentration, flow rate and pressure.

The pressure drop can be modelled according to Eq. (13), where $k_{fric}$ is the friction coefficient.

13 $$\frac{dP}{{dz}} = \frac{{ - k_{fric} \mu v}}{{d_{H}^{2} }}$$

At the retentate side, the flow rate changes, according to Eq. (14), where $z$ is the distance within the flow path of the fluid and $w$ is calculated by dividing the active area of the membrane module by its length.

14 $$\frac{{dF_{h} }}{dz} = - wJ_{w}$$

At the retentate side, the concentration changes according to Eq. (15), where $J_{s}$ is the diffusive flux of salt from the higher concentration side to the lower concentration side of the active layer, as described by Eq. (16).

15 $$\frac{{dC_{b,h} }}{dz} = \frac{{w\left( {C_{b,h} J_{w} - J_{s} } \right)}}{{F_{h} }}$$

16 $$J_{s} = B\left( {\frac{{C_{b,h} exp^{{\left( {J_{w} /k_{h} } \right)}} - C_{b,l} exp^{{\left( { - J_{w} K} \right)}} }}{{1 + B\left( {exp^{{\left( { - J_{w} K} \right)}} - 1} \right)/J_{w} }}} \right)$$

Equations 17 and 18 illustrate the rates of changes of flow and concentration in the permeate side of the membrane.

17 $$\frac{{dF_{l} }}{dz} = - wJ_{w}$$

18 $$\frac{{dC_{b,l} }}{dz} = \frac{{w\left( {C_{b,l} J_{w} - J_{s} } \right)}}{{F_{l} }}$$

If a co-current system is to be simulated, instead of counter-current, $F_{l}$ must adopt a negative sign in both Eqs. (17) and (18). This is because, unlike fluid velocity, flow rate is a scaler and could not adopt negative values.

## Novel RO separation scheme

**Figure 2:**
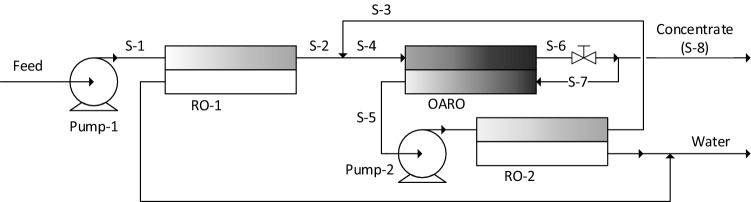
The novel process flow diagram, investigated in this publication.

Here, the novel design is described. It includes three stages RO-1, RO-2 and OARO, as shown in Fig. 2. RO-1 is a conventional RO system. The pressure delivered by Pump-1 dictates the maximum concentration that RO-1 could deliver. All three stages are simulated to operate under retentate pressure of approximately 50 bar. The OARO and RO units can be simulated to lose 0.3 and 0.2 bar respectively, using Eq. (13). RO-1 is designed with a total membrane area, at which adding to the area could not meaningfully add to the separation.

The outlet from RO-1, is fed to the OARO unit for further water removal. No further separation could occur in the OARO unit, without dosing some solution into the permeate side, to provide osmotic assist. Therefore, a fraction of the stream, S-6 is split and fed to the permeate side, to reduce the osmotic pressure difference, resisting the water flux.

In the OARO unit, the retentate and the permeate flow in opposite directions; this is known as counter-current flow. This flow mode ensures that the retentate is provided with the highest level of osmotic assist, where the retentate concentration is at its highest. This phenomenon is shown to provide product concentrations much higher than possible, with a single RO unit, in the next section.

RO-2 is a conventional RO system, and it concentrates S-5 to ensure that S-2 and S-3 have equal concentrations. Without RO-2, the two merging steams would have different concentrations, which would waste osmotic potential and energy^32,56^.

The OARO part of the design has been proposed in flow diagrams patented by Blohm et al.^11^. The novel improvement, in Fig. 2 is the energy saving mechanism added, via the RO-2 system.

## Results and discussions

### Model fitting for reverse osmosis

Lee and Kim^35^ published experimental data, for the reverse osmosis of aqueous sodium acetate. These are graphs of flux values and their corresponding hydraulic pressures and concentrations. The data extracted from their graphs, are shown in Table 2.

**Table 2:** The feed side concentration, flux and hydraulic pressure, by Lee and Kim^35 ^— Permeate concentration ≅ 0.

Concentration (mol/L)	Measured flux (µm/s)	Hydraulic pressure (MPa)
0.00	5.50	0.4
0.02	4.15	0.4
0.04	2.60	0.4
0.06	1.10	0.4
0.08	0.45	0.4
0.00	6.95	0.5
0.02	5.10	0.5
0.04	3.60	0.5
0.06	2.45	0.5
0.08	1.30	0.5
0.10	0.50	0.5
0.00	8.10	0.6
0.02	5.30	0.6
0.04	3.75	0.6
0.06	2.45	0.6
0.08	17.0	0.6
0.10	0.85	0.6
0.12	0.40	0.6

Before the flux model is used to compare against the experimental flux values of Table 2, the mass transfer coefficient is estimated by the empirical film-model correlation, as laid out in “Empirical mass transfer coefficient calculations” section, using the membrane and fluid characteristics, listed in Table 3.

**Table 3:** Assumptions, used to estimate the flux in the RO modules.

Parameter	Units	Value	Reference
$\rho$	kg/m^3^	997	Assumed that of water
$\mu$	Pa s	0.001	Assumed that of water
$d_{H}$	m	0.001	Typical according to Henley et al.^26^
$L$	m	1	Typical for industrial modules
$D$	m^2^/s	1.089 × 10^–9^	^34^
$A_{M}$	m/s/Pa	1.45 × 10^–11^	^35^

**Figure 3:**
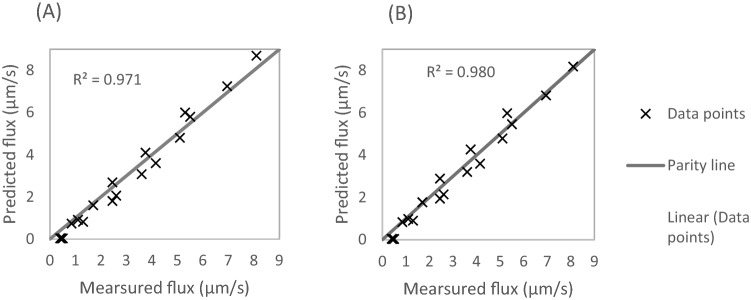
Parity line of simulated flux plotted against the measured flux values, from Table 3. (**A**) Model constants are determined from Sherwood number. (**B**) Model constants are determined from RMS fitting to the experimental data.

Using the parameters listed in Table 3, the value of 6.82 × 10^–6^ m/s is calculated, for $k = k_{h}$. The value of 1.45 × 10^–11^ m/s/Pa has been used, for $A_{M}$, as published by Lee and Kim^35^. Equation 6 is then used to predict the flux values in Table 2. The simulated flux values are plotted against the measured flux values, in a parity line graph, as shown in Fig. 3A.

Despite the slight scatter in the data, Fig. 3A boasts a good fit, and the model seems to follow the data trend very well. Root Mean Squared (RMS) fitting is also conducted on the flux values of Table 2, and the results are shown in Fig. 3B. The RMS fitting results show a marginal improvement compared to the empirical method. Its corresponding RMS error is also marginally smaller, as shown in Table 4. The following section uses the RMS fitting results to make predictions.

**Table 4:** Comparison of the two methods for determining the constant of the flux model.

Method of model construction	$k$ (m/s)	$A_{M}$ (m/s/Pa)	RMS error for $J_{w}$ (%)
Empirical film-model correlation	6.82 × 10^–6^ m/s	1.45 × 10^–11^	14
RMS fitting results	9.48 × 10^–6^ m/s	1.36 × 10^–11^	12

### Model fitting for osmotically assisted reverse osmosis

Askari et al.^3^ tailor-made a prototype hollow fiber membrane, for OARO. They tested the membrane by maintaining identical concentrations at both the permeate and the retentate chambers and measuring the flux under 30 bar hydraulic pressure. They did so, for sodium chloride molarities of 0.035, 0.6 and 1.2, and reported pressure-specific water flux values of 2.2, 0.4 and 0.15 L/m^2^/h/bar, respectively. The flux model as described in Eq. (7), is fitted to these three data points. Once the model is validated experimentally for sodium chloride, the model constants can be adjusted for sodium acetate.

**Figure 4:**
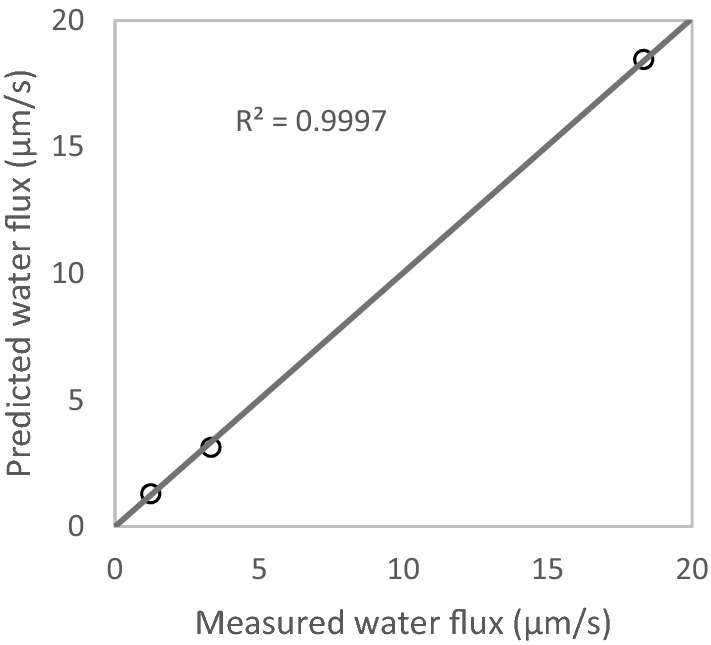
Parity line graph of simulated flux plotted against the measured flux — OARO experiment by Askari et al.^3^, under 30 bar hydraulic pressure, with equal permeate and retentate bulk concentrations of 0.035, 0.6 and 1.2 molar.

Salt permeability of 1.1 × 10^–7^ m/s is used^31^. The fiber tubes’ internal diameter is 324 µm^3^. The $k_{h}$ value for sodium chloride is estimated to be 2.5 × 10^–5^ m/s, as explained in “Empirical mass transfer coefficient calculations” section. $K$ is estimated to be 423,000 s/m and $A_{M}$ is estimated to be 2.51 L/m^2^/h/bar by RMS fitting of the model to the three data points by Askari et al.^3^. The model predictions are plotted against their corresponding flux measurements, as shown in Fig. 4, where the model demonstrates a good fit to the data.

The product of $K$ and diffusion coefficient is often reported in the literature, as the structure number of the membrane^44^. This is a property of the membrane and does not depend on the solute. For the OARO membrane tested by Askari et al.^3^, the structure number is estimated to be 701 µm.

$K$ is then calculated to be 644,000 s/m for sodium acetate, by dividing the structure number of the membrane by the diffusion coefficient of sodium acetate. The $k_{h}$ value for sodium acetate is estimated to be 1.9 × 10^–5^ m/s, as explained in “Empirical mass transfer coefficient calculations” section. These model constants pertaining to sodium acetate are then used to simulate the OARO part of the design.

### Simulation of the case study

In “Model fitting for reverse osmosis” and “Model fitting for osmotically assisted reverse osmosis” sections, the model constants are determined and validated against laboratory data. They are used, in this section, to simulate the design, depicted in Fig. 2. The design bases are provided in Table 5, where the baseline throughput corresponds to 1 kg/s of sodium acetate.

**Table 5:** Bases of the case study for the concentration of dilute aqueous sodium acetate.

Length of membrane modules (m)	1
Feed concentration (wt%)	1
Target product concentration (wt%)	30
Dilute feed flow rate (kg/s)	100
Hydraulic pump pressures (bar)	≅ 50

**Figure 5:**
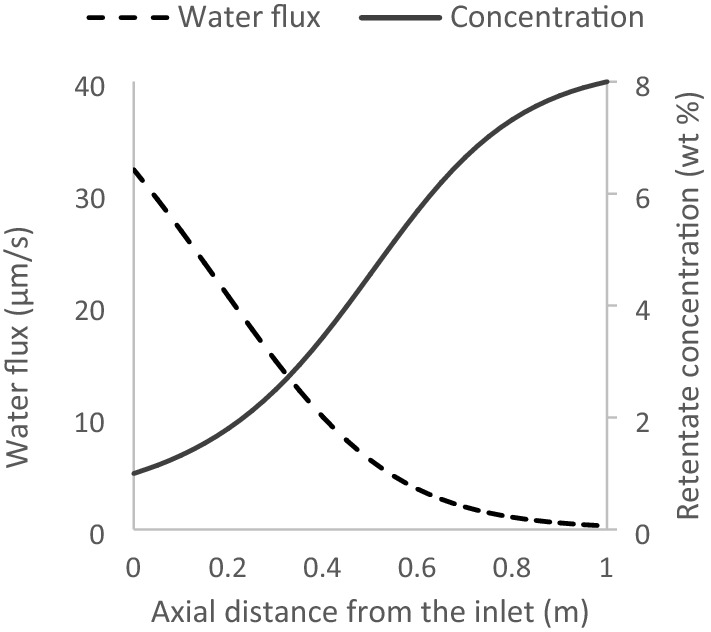
Concentration and flux profiles along the length of the modules, for RO-1, corresponding to the bases of the case study, in Table 5.

The membrane area of 8000 m^2^ is simulated to provide 8 wt%, in the stage RO-1 of Fig. 2. The water flux and retentate concentration profiles, corresponding to this value of total membrane area, are shown in Fig. 5. The flux diminishes towards the end of the flow path, indicating that little more concentration could have been obtained by adopting a higher membrane area.

An example of a membrane module, considered for the case study, is the model JSW-8040-HF, manufactured by Shandong Jozzon Membrane Technology Co., Ltd. These modules can withstand up to 69 bar of hydraulic pressure and pH values ranging between 3 and 10. They provide a membrane area of 35.2 m^2^, per module^30^. 227 of these modules, installed in parallel, would provide, approximately, the area simulated for the RO-1 section of the design.

**Figure 6:**
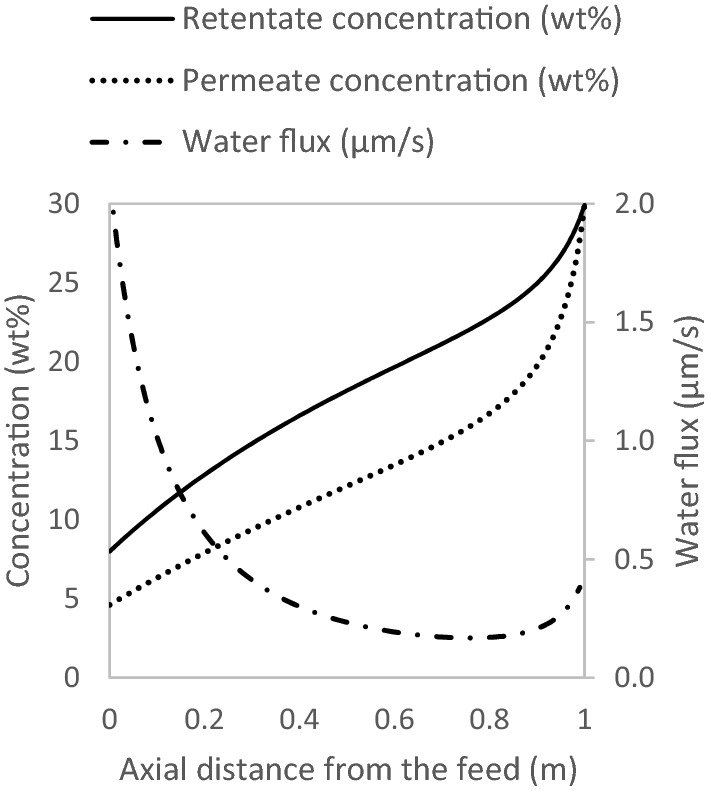
Concentration and flux profiles along the length of the modules, for OARO, corresponding to the basis of case study laid out in Table 5 — feed at 8 wt%.

The OARO system is simulated to further concentrate the 8 wt% solution to 30 wt%. This is not concentrated enough, to cause crystallisation and fouling or blockage of the membrane module^25^. The concentration and flux profiles are shown in Fig. 6.

The horizontal axes, in Figs. 5 and 6 represent $z$, in the mass balance equations. At $z$ equal to the full length of the modules, the concentration is at its highest, for both RO and OARO. However, the flux at this point is at its lowest, only for RO (see Fig. 5). For OARO, at $z$ equal to the full length of the modules, the retentate and permeate have identical bulk concentrations, which boosts the flux. This boosted flux diminishes the osmotic assist, a short distance from the full length of the modules. Thus, in the case of counter-current OARO, the lowest flux occurs near the full length of the modules.

The steady state simulation of the OARO unit, with the two recycle streams, S-7 and S-3, as shown in Fig. 2, can be achieved. It requires an iterative method, as follows: Initially, the OARO system is simulated, assuming that S-3 is not merged with S-2 (i.e. S-2 and S-4 are the same stream). The OARO unit is simulated, based on arbitrary values of the total active membrane area and the flow rate and concentration of S-5. These three arbitrary values are then adjusted iteratively, so that both S-6 and S-7 have concentrations of 30 wt% and the flow rate of S-3 is equal to half that of S-4.

This initial simulation provides membrane area of 22,500 m^2^. Since S-3 and S-2 have been simulated to have the same concentration and flow rates, the actual throughput for OARO is twice the throughput, used in the initial simulation. The recycling of S-3 can be accounted for, by doubling the simulated areas for OARO and RO-2. The simulated flow rates of S-3, S-5, S-6, S-7 and S-8 are doubled, for the same reason. Since S-3 has the same concentration as S-2, recycling it does not change the concentration of the ingress to the OARO unit. Therefore, the flux and concentration profiles in the OARO unit remain unchanged, which makes the above extrapolation possible. The final simulation results are summarised in Table 6.

**Table 6:** Simulation results for the case study, described in “Novel RO separation scheme” section.

Membrane system			RO-1	OARO	RO-2
Membrane area (m^2^)			8000	45,000	4200
Stream flow rate (L/s)	Retentate	Inlet	100.3	25.0	21.1
		Outlet	12.5	5.2	9.4
	Permeate	Inlet	0	1.8	0
		Outlet	87.8	21.7	9.6

### A demonstration of the energy saving potential

Sodium acetate is generally sold in pure solid form, rather than a concentrated solution. In this section, the heat drying of the solution is considered to produce sodium acetate powder, for three scenarios: In scenario 1, heat is used for the drying of the 1 wt% stream, without any membrane separation. In scenario 2, the concentration is brought from 1 wt% to 8 wt%, in a conventional RO system, followed by heat drying. In scenario 3, the novel scheme, depicted in Fig. 2, brings the concentration from 1 to 30 wt%, followed by heat drying. In each scenario, the projections are based on 1 kg of sodium acetate, produced. The dryer heat is estimated based on the latent heat of evaporation of 2.26 MJ/kg^17^. The pump work is estimated, based on differential pressure of approximately 50 bar and 80% pump efficiency. The total energy required, for each scenario, is calculated as the sum of the pump electric energy and dryer heat, as shown in Table 7.

**Table 7:** The energy requirement calculations, based on 1 kg of sodium acetate, recovered from a 1 wt% solution.

Scenario	1	2	3
Dryer feed concentration	1 wt%	8 wt%	30 wt%
Evaporated water (kg)	99	11.5	2.33
Dryer heat (MJ)	224	26.0	5.3
Total volume pumped (L)	0	100	107
Pump work (kJ)	0	627	763
Total energy required (MJ)	224	26.6	6.1

Most of the water removal is achieved in RO-1. The total energy consumption, in Scenario 3 is four times smaller than that of scenario 2. Such a significant difference had been anticipated, as evaporative water removal is widely established to consume many times more energy than osmotic desalination. Scenario 3 consumes five times less heat than scenario 2. This comes at the cost of 22% more electric power consumption and 7 times more membrane area.

## Conclusions

A novel process flow diagram is proposed, for osmotic separation of aqueous solutions, with low permeability, relative to water. The novel component is the counter-current osmotic assist, via retentate split, in conjunction with the additional RO unit that prevents the recycled draw solution from merging at dissimilar concentrations.

The internal concentration polarisation (ICP) model provides a good fit to the OARO experimental data, obtained from the literature. The novel design is simulated, using the ICP model, to concentrate the solution to 30 wt% sodium acetate, which is much higher than possible, with a typical reverse osmosis system. The use of this novel design, instead of standard RO, is simulated to reduce the total energy consumption of a sodium acetate drying system, by a factor of four.

For the novel design, the total membrane area is simulated at 57,200 m^2^. Although this is seven times higher than the standalone RO system, it saves 180 GWh/year of energy. Furthermore, the simulated water flux, in the OARO part of the design is on average 23 times smaller than the water flux in the RO part. This may allow the OARO membrane part to last longer, which would help to justify the novel design.

## Data Availability

All model constants are stated with citation, at appropriate points withing the manuscript. The experimental data, by Lee and Kim^35^, are presented in Table 2.

## List of symbols

$$A$$: Membrane area, m^2^
${A}_{M}$: Membrane permeability coefficient, m/Pa/s
${C}_{b,h}$: Retentate side bulk concentration, mol/m^3^
${C}_{b,l}$: Permeate side bulk concentration, mol/m^3^
${C}_{m,h}$: Retentate side membrane concentration, mol/m^3^
${C}_{m,l}$: Permeate side membrane concentration, mol/m^3^
$$B$$: Salt permeability, m/s
$D$: Aqueous ion diffusion coefficient, m^2^/s
$${d}_{H}$$: Hydraulic diameter, m
${F}_{h}$: Retentate flow rate, m^3^/s
${F}_{l}$: Permeate flow rate, m^3^/s
$i$: Number of moles of ions corresponding to one mole of salt dissolved, Dimensionless
${J}_{h}$: Retentate side ion flux, mol/m^2^/s
${J}_{s}$: Salt flux, mol/m^2^/s
${J}_{w}$: Volumetric water flux through the membrane, m/s
${k}_{fric}$: Friction coefficient, Dimensionless
${k}_{h}$: Mass transfer coefficient on the retentate side, m/s
$K$: ICP model constant $K=\tau {\delta }_{s}/\left(D\varepsilon \right)$, s/m
$\Delta P$: Hydraulic pressure difference across the membrane, Pa
${N}_{Re}$: Reynolds number, Dimensionless
${N}_{Sh}$: Sherwood number, Dimensionless
${N}_{Sc}$: Schmidt number, Dimensionless
$q$: Hight of the flow channels within the membrane module, m
$R$: Ideal gas law constant, J/K/mol
$S$: Membrane structure number, m
$$T$$: Absolute temperature, K
$v$: Liquid superficial velocity in the membrane channels, m/s
$$w$$: $$w=A/L$$, m
$z$: Distance from the feed inlet, within the membrane module, m
$L$: Length of the membrane = length of the membrane module, m
${\delta }_{s}$: Membrane support thickness, m
$\Delta \pi$: Osmotic pressure difference across the membrane skin, Pa
$\varepsilon$: Membrane porosity, Dimensionless
$\mu$: Dynamic viscosity, Pa s
$\rho$: Liquid density, kg/m^3^
$\tau$: Membrane tortuosity, Dimensionless
